# Carbolic Acid

**Published:** 1884-04

**Authors:** Frank French

**Affiliations:** Rochester, New York


### Carbolic Acid.

Read before the New York Dental Society, by Frank French, M. D. S., of Rochester, New York.

Tar, with what is obtained from it by distillation, is a complex product. We will only refer to two substances, viz.: Creosote, which is obtained from wood tar, and Carbolic Acid, which is obtained from coal tar; and though differing in composition, their action is very much alike.

Creosote is found in the products of dry distillation of wood, and of various vegetable as well as animal substances, and of Benzoin resin, used in fumigation. Tar barrels, which have been burned in time of epidemics to purify the atmosphere, give out this substance, but would give a greater quantity if the flames were suppressed, and distillation only allowed.

The world has admired this substance without knowing of its existence: has sought for it, using various names to express it; wrapping it in bundles to carry around them, burning it in pastiles for fumigation, and sometimes in public in great bonfires. The most civilized peoples of olden times, kept in the products of tar, the dead that they desired to preserve to a joyful uprising.

Thus we see that the antiseptic properties of tar were known at a very early period, but the process of extracting its subtle essences, and bringing them into general use, was reserved for the nineteenth century.

Creosote was discovered in 1832, by Reichenbach; it is officinally described as a peculiar substance obtained from wood tar. It is a colorless, oily liquid, of a peculiar disagreeable penetrating odor, resembling that of wood smoke, and has a burning, acrid taste, which is perceived throughout the whole extent of the buccal, nasal, and pharyngeal mucous membrane. It burns briskly in the air, emitting large volumes of smoke. It coagulates albumen, but exerts no action upon fibrin. It is sparingly soluble in water, but readily so in ether, alcohol, acetic acid, and the essential oils. It acts as a solvent of iodine, phosphorus, sulphur, and the resins. Its action, both upon animal and vegetable organisms, is so identical with that of carbolic acid, that there is no real reason for giving it the preference which some do, even if it could be obtained pure, which is nearly impossible. In fact, *pure* creosote is almost a myth. Dr. Brophy says: "Of 25 samples of creosote, purchased from as many different dealers, I find on examination that, with one exception, all contain carbolic acid; some were pure carbolic acid." This being the case, is it not preferable to use pure carbolic acid, and call it so, rather than use a mixture and call it creosote? They may be distinguished from each other by the following tests:

Carbolic acid becomes solid at ordinary temperatures, while 17° below zero does not affect creosote.

Carbolic acid is soluble in water, 1 part in 95, or 1 in 20 of boiling water, and this solution is colored a deep violet purple by the addition of a solution of ferric chloride. Creosote is soluble in water, 1 part to about 125, and is colored brown by ferric chloride. An alcoholic solution of carbolic acid is colored a light olive green by ferric chloride; an alcoholic solution of creosote, a dark sea-green. Carbolic acid forms a jelly when shaken with collodion creosote does not.

Carbolic acid was discovered by Runge, in coal tar, in 1834, and given the name it now bears. It is procured from coal tar by distillation, at a temperature of from 300° to 400° Fah. In 1841 it was investigated by Laurent, who gave it the name of Hydrated Oxyde of Phenyl: it was afterwards called Phenic Acid, and, as it is closely related chemically with the alcohols, it was called by some Phenylic Alcohol. It is, however, best known by the name given it by its discoverer.

Before its use separately, its virtues were exhibited in the crude material known as coal tar, in 1844, by M. Bayard. About 1860, Kuchenmeister used it under the name of "Spirol," both in medical and surgical practice, and as a means of arresting putrefaction and preventing the development of fungi. In 1863, Dr. Lemaire issued a treatise upon Phenic Acid, illustrating its power of preventing fermentation and putrefaction, and its application to medical and surgical uses. In 1865, M. Declat illustrated the power of the acid to destroy the microphytes and microzoaires, developed during fermentation and putrefaction, and of preventing the changes which constitute these processes. After this it came into general use, both in this country and Europe. Its vogue is chiefly to be ascribed to Prof. Lister, who commenced its use as a surgical dressing in 1865.

Carbolic acid, in its pure state, is solid at ordinary temperature, and is either in acicular crystals, or crystalline masses; colorless when pure, but when impure a reddish brown, or becoming so on exposure to light and air, which is said to be caused by the presence of xylic and cresylic alcohols, which always accompany it in its impure state, and adhere to it tenaciously. On exposing the crystals to the air they attract moisture and deliquesce. It becomes liquid by the presence of water in very small quantities.

With regard to its solubility in water, there is a great diversity of opinion in regard to the proportions. It is claimed to be soluble in 15 per cent. of water, and from that, all the way up to 95 per cent. I find, by experiment, that the crystals will absorb 10 per cent. of water by weight, and become perfectly liquid. After that proportion of water has been taken up, there is no union of the acid and water until 95 per cent. of water has been added, at which point the acid is entirely dissolved, but not before. It is, however, perfectly soluble in 20 per. cent. of boiling water. It is very soluble in ether, alcohol, acetic acid, glycerine, and the fixed oils. It is inflammable, burning with a reddish flame and dense volume of smoke; it melts at from 95° to 160° and boils at from 359° to 370° Fah. Nitric acid converts it into picric acid, and it is largely used in its manufacture. It reduces many metallic salts, especially those of silver and copper; with alkalies it forms acid salts, and combines readily with iodine, bromine, quinine, etc. As generally sold, if pure, it is an oily, colorless liquid, with a strong smell like creosote, and having like it a pungent acrid taste. With regard to the propriety of calling it an acid, it does not respond to the usual test with litmus paper, when tested at full strength, but in a solution of 5 per cent. carbolic acid and 95 per cent. of water, we get a decided acid reaction, coloring blue litmus paper red, almost instantly. Why the addition of water should change a neutral solution to an acid, I am unable to state. I discovered this acid property accidentally, about a year ago, while making some experiments. It may have been known to others, but I have seen no account of it.

As a therapeutic agent it is invaluable, as it possesses stimulant, narcotic, irritant, styptic, antiseptic, and escharotic properties. It unites with albumen and gelatine, forming with them insoluble compounds. It has remarkable antiseptic powers, and consequently is a valuable topical application, in cases attended with offensive purulent or other discharges, but it is not a disinfectant, as generally considered. It prevents decomposition, but does not destroy or render inoffensive the products of decomposition. It is commonly used as a deodorizer, but it is not one; it has *no* action upon foul emanations. Its operation consists in preventing their generation, by arresting putrefactive changes in the substances which exhale them. Lemaire claims that these changes are due to septic infusoria in the atmosphere, which, "when admitted into wounds, ulcers, etc., produce a decomposition in the part and its secreted fluid, aiding the formation of pus; that this decomposition is effected by a vital action, similar to the production and multiplication of organisms in fermentations; that carbolic acid in small doses has the power of preventing and arresting any such decomposing effects from these organisms, by at once and immediately destroying the life of the organisms themselves."

Whether this view be correct or not, it is undeniable that it will prevent putrefaction, or arrest it where it has already begun, and when putrefaction ceases, the foul emanations cease also, and this is, undoubtedly, the way it works when applied to drains, sewers, etc., and decayed substances, whether animal or vegetable.

All the lower forms of vegetable organic life are destroyed by carbolic acid. It arrests fermentation, destroys mould and mildew, and prevents the germination of seeds in water containing a 100th or 200th part of this acid. A strong solution applied to the roots of trees and shrubs kills them, and, if to the flowers, leaves, and fruit, these die.

On animal organisms a very small proportion of it in water containing bacteria, amoeba, and other infusoria, speedily destroys them. It is equally destructive to the life of ascarides, earthworms, caterpillars, fleas, moths, ants, and other insects. Fish, frogs, and leeches, die speedily in a solution of it, and two or three drops applied under the wing of a sparrow, produce convulsions and death. In the case of dogs, a 5 per cent. aqueous solution administered, causes them to fall, cough, become convulsed, and then paralyzed in both sensation and motion; the acid is exhaled in breathing, and they gradually recover. One-half fluid drachm dissolved in twelve fluid drachms of glycerine, and thrown into the stomach of a dog, caused all the above symptoms, and, in addition, a bloody froth was ejected, and death ensued suddenly in ten minutes after administration. After the death of animals from poisonous doses, extreme congestion is found of the brain and its membranes, alimentary canal, and the lungs. The blood is not coagulated. On man, when six or eight grains in a wine-glass of water are taken, a sense of numbness is felt on the lips and in the mouth, followed by a sensation of coolness. If the stomach is empty, slight nausea, and an uneasy sensation in the abdomen follows, with vertigo, singing in the ears, and slight deafness. When swallowed by accident, or design, the mucous membrane appears as if brushed over with a strong solution of nitrate of silver, and becomes hard and dry, like leather. This change in the condition of the mucous membrane, is due to the power of carbolic acid to coagulate the albumen of the tissues. From medicinal doses, a cooling, grateful effect is felt in the stomach. Cases of poisoning have resulted from local applications. The warnings of danger, which may be expected when the remedy is brought in contact with the tissues at any point, are, besides the local irritation, sudden vertigo, contracted pupils, pallor of the face, difficult respiration, and feeble circulation.

When the dose is a fatal one, unconsciousness quickly follows; the breathing becomes stertorous, the surface grows cold, the action of the heart more and more feeble, and death finally occurs from failure of respiration. Convulsions occur in animals, but in man this symptom is lacking. The poisonous effects of carbolic acid are remarkably uniform for the same dose. An ounce, or more, taken by an adult, produces an intense burning pain in the throat and œsophagus, followed by staggering as from intoxication, and then complete insensibility. The face is pale, lips and hands livid; the pulse, sometimes above and sometimes below the normal rate, is soft and compressible; respiration stertorous, tongue swollen, and a discolored foam on the lips. The skin, breath, and urine, smell strongly of carbolic acid. Death has occurred in a few minutes; in most of the fatal cases in from one to three hours, and they hardly ever live two days. It affects the urine, even when applied locally, and one of the earliest indications of toxic action is a dark greenish, blackish or smoky hue of the urine. Stadeler claims to have discovered carbolic acid in normal urine, but this is disputed. In cases of poisoning, the stomach should be emptied as soon as possible, and albumen in the form of fresh eggs, largely diluted with cold water or milk, freely administered. Saccharate of lime is a chemical antidote, and in cases of poisoning may be used freely. Oils and glycerine should not be given, because they favor absorption, but vegetable demulcents used freely, in order to protect the mucous membrane as much as possible. Atropia is a physiological antagonist of great value. The application externally has sometimes been fatal. Three women used crude carbolic acid for itch, mistaking it for sulphur ointment, applying it all over the body with a sponge. One died in three hours, another in two days, and the third recovered after an illness of three weeks. Another case of a female child, who sat down upon a block which had been freely sprinkled with a solution of an ounce of acid to one quart of water. Blistering, followed by ulceration of the labia and buttocks, took place, and the child died on the tenth day.

For internal use, the dose is from one-fourth to two grains, in a wine-glass of water. One or two drops in a glass of water will arrest nausea and vomiting. Cholera morbus and cholera infantum are frequently checked by its exhibition. Eructations of gas, and flatulence, due to fermentation of food, are arrested by it. Inhalations of carbolic acid spray, of the strength of 1 per cent., are used in chronic nasal catarrh, hay fever, and whooping-cough, which is said to be due to the fact that it destroys the minute organisms which cause the morbid action in these maladies. Schnitzler, of Vienna, employed the subcutaneous injection of it in more than one hundred cases of consumption, using from one-eighth to one-fourth of a grain once, and, in some cases, twice daily. In many cases the fever lessened, pulse became slower and stronger, and night sweats diminished.

Based on the germ theory of disease, it has been used with some success in typhoid fever, diphtheria, scarlet fever, and erysipelas. In parasitic skin diseases a weak solution is very serviceable. It is largely used in surgery in carrying out the antiseptic system of Prof. Lister, who, believing that suppuration, pyœmia, and various other troubles connected with open wounds, arise from the irritation of minute germs contained in the air, devised a process by which the air, before reaching the raw surface, is filtered through carbolic acid, and thus deprived of its irritating properties. He prevents the access of air during operations, by surrounding the part with an antiseptic atmosphere, composed of a sprayed watery solution of carbolic acid, 1 to 40. The instruments and fingers of the surgeon are washed with carbolized oil, and the arteries tied with carbolized catgut ligatures. By adopting these precautions, and the careful dressing of wounds, he has obtained excellent results, not only after ordinary operations, but in chronic abscesses, and various diseased conditions connected with the joints.

Strong carbolic acid, applied to the surface of healthy skin, causes an immediate tingling and burning sensation; the spot becomes white, and the skin around it is tinged with red; the symptoms are similar to those resulting from the application of a powerful caustic. In a few days there is exudation from the surface, sometimes dry and scaly, but frequently there is formation of pus. Dr. Bill was the first to demonstrate the anæsthetic properties of carbolic acid. He states that "by applying a solution of it to the integument, insensibility to pain may be sufficiently induced to permit free incisions without suffering." It coagulates blood, but does not stop bleeding. In solution, it coagulates albumen, arrests fermentation, instantly destroys the lower forms of animal and vegetable life, and in very small proportions prevents mouldiness in vegetable juices, and protects animal substances from putrefaction. In a solution of 1 to 500 it destroys vegetable mould, and is equally destructive to minute organisms. According to Dr. Neuman a solution of 1 per cent. is sufficient to destroy bacteria. It was mentioned here, last year, that bacteria had been seen crawling over crystals of carbolic acid. I think the crystals, like those of many other acids, are inert, and must be in solution to produce an effect. It is very certain in many cases we get better results from a diluted solution than from full strength.

It seems to have the effect of promoting growth of healthy granulations, and hastening healing processes of wounds. It relieves pain without causing inflammation and arrests suppuration. Its application should be repeated as long as pus is formed on the surface of the wound, but should not be applied so long as the eschar from the former application remains attached. It is an irritant, and if applied long enough to the skin will cause sloughing. It is an escharotic, and if applied to the integument or mucous membrane, produces a burning sensation of short duration, and there is formed a whitish superficial eschar, said to be due to the coagulation of albumen, which subsequently becomes brownish and drops off. As regards the strength in which carbolic acid should be used, it must be governed by the exigencies of the case, and the judgment of the operator. For external use where escharotic action is desired, as in gangrenous or specific ulcers, it may be used in the strongest liquid form; but for ordinary skin diseases, a solution of from 1 to 5 per cent. is sufficient, also where a gentle stimulation is desired in order that the parts may take on recuperative action. Where prompt, decisive action is required, and heroic treatment demanded, it may be used full strength, but where only gentle stimulation is desired, a 5 per cent. solution is sufficient. Ulcers and suppuration, whether on the outer surface or from the mucous passages, are benefited by its use, and fistulous ulcers, especially those connected with the mouth, are generally controlled by it. It has been highly recommended as a dentifrice in carious teeth and offensive breath. On account of its anæsthetic properties it relieves pain caused by an exposed pulp almost instantly, and is useful to apply to the pulp before applying arsenic, when it is necessary to destroy it. Some good operators claim that they are able to extirpate the pulp alive, almost painlessly, by working carbolic acid in between the pulp and walls of the canal with a fine broach.

Where the nerve is exposed, and it is desirable to save it alive, it should never be used full strength, as its escharotic action may cause sloughing, and being confined under the material used for capping, the death of the entire nerve is apt to follow; but a solution of 1 to 5 per cent. may be used with great benefit in such cases, as it is a gentle stimulant, slightly antiseptic, and will not cause sloughing and death.

Applied in full strength to a cavity just prepared for filling, a painful burning sensation is experienced for an instant, and the cavity is not only less sensitive, but that portion of the tooth seems to lose a portion of its conductivity, so that it is less sensitive after being filled than it would be if it were not used. This is said to be due to its coagulating the contents in the ends of the freshly cut tubuli, and thus breaking the connection of conductive power between the filling and the nerve. It is also claimed that its use in cavities before filling, renders inert any small particles of decayed matter that might possibly remain. Brophy says: "It is a most excellent remedy to apply to carious teeth during the process of excavating, to enable us to more clearly see the extent of the caries; in many cases we are surprised, upon moistening the cavities with it, to find carious tissue, which, if permitted to remain, would soon render another operation necessary."

Sensitive cavities are rendered much less so by applying carbolic acid, full strength, then inserting a gutta-percha filling; allowing this to remain a few days, the cavity may be excavated with very little pain. It can be used in this way as an obtunder without endangering the pulp.

It is an excellent dressing for nerve canals after removing the pulps, as it closes the ends of the tubuli by coagulating the contents; but as in destroying the pulp we sometimes destroy the contents of the ends of the tubuli, or the ends of the so-called soft fibres of the Tomes, it would be safer to slightly enlarge the canal so as to reach what has been called the "dead line," before applying the carbolic acid.

It is the custom of many operators before filling nerve canals to close the apicial foramen with a small pledget of cotton, saturated with carbolic acid, on account of its antiseptic properties. This is condemned by some, but where carefully done, the results are almost uniformly good. It has been claimed that a saturated solution of iodine in creosote or carbolic acid has been successfully used in cases where there is wasting of the gums and processes, with exudation of pus and loosening of the teeth, changing the pus-producing to a plasm-producing surface. It stimulates those parts which are weak, but still have vitality enough to be restored, and destroys those which have not. It should not be applied oftener at first than once a day, and after a few applications once in two or three days. It should be applied carefully, to avoid coming in contact with healthy parts, and covered with a saturated solution of glycerine and tannin.

In the treatment of nearly all cases of abscess, where it is not accompanied by necrosed bone, carbolic acid may be regarded as nearly a specific, but where necrosis is present other treatment is indicated. In using it where it comes in contact with the skin, its caustic action can be overcome by rubbing the parts with oil. In the treatment of alveolar abscess, the dentist comes to regard it as a sheet-anchor. Many other remedies have been used, but none of them seem to take its place. There is a diversity of opinion concerning the strength of the solution to be used in such cases. Where there is a fistula I always use full strength, pumping it with a piston through the root, until it comes through the fistula clear and free from pus. If necessary I repeat it in two or three days, but rarely have to use it more than twice. It should not, however, be used until a disinfecting agent has been applied in the root, otherwise the effete matter in the tubuli will be coagulated, and remain to give rise to trouble through the tubuli into the lacunæ and thus through the cementum. These are dead men that sometimes will not rest quiet in their graves.

The uses of carbolic acid are so numerous as to make it impossible to refer to more than a few in a paper of this kind. In very small quantities it is azymotic, *i. e.*, fatal to all the lower orders of animal and vegetable life; it is also antiseptic, *i. e.*, opposed to putrefaction or decay, even preserving the organisms which it kills. By good authorities all epidemic, contagious, and infectious diseases are believed to be zymotic, *i. e.*, of the character of a fermentation dependent upon living organisms; now as all the processes of decay and putrefaction are dependent upon fermentations, which are caused and kept up through the agency of cell-life, or organisms of a low vitality, in fact are also zymotic, a good key to its powers and uses is at once obtained, and at the same time a good guard against its misapplication.

The hurtful material in foul emanations and foul air may be inodorous and insensible, but is endowed with vitality, and the laws of nature tend to direct the material to a soil and climate fitted for its functions of germination and reproduction. If the azymotic could be distributed in the same way, there would soon be an end of zymotic disease, but with it an end of all fermentation and an end of the present order of creation, in which fermentation performs an indispensable part.

Since writing this paper I have been informed by Dr. T. H. Smith, of Bloomington, Ill., that common vinegar is a perfect antidote for the action of carbolic acid, internally as well as externally. I have had no opportunity to verify his statement with regard to its internal action, but in several cases externally it has been applied with the most satisfactory results, stopping the action of the carbolic acid at once, and no unpleasant effect following.

